# PTPN2 improved renal injury and fibrosis by suppressing STAT‐induced inflammation in early diabetic nephropathy

**DOI:** 10.1111/jcmm.14304

**Published:** 2019-04-06

**Authors:** Ya Li, Huimin Zhou, Yulin Li, Lu Han, Ming Song, Fangfang Chen, Guokai Shang, Di Wang, Zhihao Wang, Wei Zhang, Ming Zhong

**Affiliations:** ^1^ The Key Laboratory of Cardiovascular Remodeling and Function Research, Chinese Ministry of Education, Chinese Ministry of Health and Chinese Academy of Medical Sciences, The State and Shandong Province Joint Key Laboratory of Translational Cardiovascular Medicine, Department of Cardiology Qilu Hospital of Shandong University Jinan Shandong China; ^2^ Department of General Practice Qilu Hospital of Shandong University Jinan Shandong China; ^3^ Department of Geriatric Medicine, Qilu Hospital of Shandong University Key Laboratory of Cardiovascular Proteomics of Shandong Province Ji’nan China

**Keywords:** diabetes nephropathy, fibrosis, inflammation, PTPN2, STAT1/3

## Abstract

Diabetic nephropathy (DN) is a chronic inflammatory disease triggered by disordered metabolism. Recent studies suggested that protein tyrosine phosphatase non‐receptor type 2 (PTPN2) could ameliorate metabolic disorders and suppress inflammatory responses. This study investigated PTPN2's role in modulating DN and the possible cellular mechanisms involved. In a mouse model combining hyperglycaemia and hypercholesterolaemia (streptozotocin diabetic, ApoE^‐/‐^ mice), mice showed severe insulin resistance, renal dysfunction, micro‐inflammation, subsequent extracellular matrix expansion and decreased expression of PTPN2. We found that mice treated with PTPN2 displayed reduced serum creatinine, serum BUN and proteinuria. PTPN2 gene therapy markedly attenuated metabolic disorders and hyperglycaemia. In addition, PTPN2 gene transfer significantly suppressed renal activation of signal transducers and activators of transcription (STAT), STAT‐dependent pro‐inflammatory and pro‐fibrotic genes expression, and influx of lymphocytes in DN, indicating anti‐inflammatory effects of PTPN2 by inhibiting the activation of STAT signalling pathway in vivo. Furthermore, PTPN2 overexpression inhibited the high‐glucose induced phosphorylation of STAT, target genes expression and proliferation in mouse mesangial and tubuloepithelial cells, suggesting that the roles of PTPN2 on STAT activation was independent of glycaemic changes. Our results demonstrated that PTPN2 gene therapy could exert protective effects on DN via ameliorating metabolic disorders and inhibiting renal STAT‐dependent micro‐inflammation, suggesting its potential role for treatment of human DN.

## 1 INTRODUCTION

Diabetic nephropathy (DN), which is characterized by glomerular and tubular disorders, is a major micro‐vascular complication^1^ and accounts for more than 50% of all cases of end‐stage renal disease (ESRD).^2^ Once early DN (stage 3 nephropathy) has developed into phase clinical albuminuria (stage 4 nephropathy), kidney failure is unavoidable and irreversible, requiring renal replacement therapy.^3^, ^4^ Hence, early treatments in stage 3 DN remain the last line of defence against kidney failure. Although strict restriction of glycaemia, blood pressure and lipids can relieve DN symptoms, current treatments are insufficient to efficiently prevent the progression of DN from stage 3 to stage 4.^5^ Hence, there is an urgent need for new strategies that interrupt mechanisms underlying the progression of stage 3 DN induced by hyperglycaemia.

Hyperglycaemia‐induced inflammation pathways play central roles in the progression of stage 3 DN.^6^, ^7^, ^8^ The major clinical and recognized hallmark of stage 3 DN is elevated albuminuria secretion (20 μg/min < urine albumin excretion rate < 200 μg/min).^9^ The classical histological features of stage 3 DN include mesangial expansion, glomerular basement membrane thickening, tubule‐interstitial fibrosis and lymphocytes influx.^10^ Recent findings suggest that the basic underlying mechanisms of DN involve high‐glucose (HG) induced production of inflammatory mediators in glomerular and tubular cells, which triggers lymphocytes infiltration, renal cell proliferation and extracellular matrix expansion.^11^, ^12^ Thus, there is a obvious need for new strategies that simultaneously ameliorate systemic metabolism disorder and renal micro‐inflammation to slow the decline of renal function in DN.

Protein tyrosine phosphatase non‐receptor type 2 (PTPN2) might be the key regulator that controls metabolism and micro‐inflammation.^13^, ^14^, ^15^ PTPN2, one of 17 intracellular and non‐receptor PTPs, was originally cloned from a human T‐cell cDNA library.^16^ It is ubiquitously expressed (eg intestinal and renal epithelium, fibroblasts, hepatocytes).^17^ It has been indicated that PTPN2 is indispensable for maintaining metabolic homeostasis,^13^ and liver‐specific PTPN2 deficiency promotes hepatic steatosis, obesity and insulin resistance (IR).^15^ Evidence is emerging for the involvement of PTPN2 in the onset and progression of inflammatory diseases, such as Crohn's disease, T1DM and rheumatoid arthritis.^14^, ^16^, ^18^, ^19^ Although these results suggest the important role of PTPN2 in metabolic diseases and inflammation, it remains unknown whether PTPN2 contributes to the progression of DN. HG‐induced STAT activation contributes to the expression of pro‐inflammatory and pro‐fibrotic factors in glomerular and tubular cells and infiltration by circulating inflammatory cells, which amplifies and perpetuates the inflammatory process in the kidney, finally resulting in the development and progression of DN.^20^, ^21^, ^22^ PTPN2 is the key negative regulator that controls the magnitude and duration of STAT signalling through several mechanisms, including kinase inhibition and STAT binding.^23^, ^24^ Thus, PTPN2 may suppress the expression of pro‐inflammatory cytokines via inhibiting STAT signalling pathway and thus ameliorate renal injury and fibrosis in DN.

In the present study, we constructed an early DN ApoE^‐/‐^ mouse model. We aimed to explore whether PTPN2 gene therapy could improve DN by regulating systemic metabolic disorders and inhibiting local inflammation in kidney.

## 2 MATERIALS AND METHODS

### 2.1 Reagents and antibodies

Dulbecco's modified Eagle's medium (DMEM) and foetal bovine serum (FBS) were from Gibco (Grand Island, NY). Streptozotocin (STZ) was obtained from Sigma Chemical (St. Louis, MO). Primary antibodies for immunohistochemistry and Western blot analyses included PTPN2 (ab180764; Abcam); P‐STAT1 (#9167; Cell Signaling); P‐STAT3 (#9145; Cell Signaling); Arginase I (16001‐1‐AP; Proteintech Group Inc); Arginase II (14825‐1‐AP; Proteintech Group Inc); CD11c (17342‐1‐AP; Proteintech Group Inc); CD206 (18704‐1‐AP; Proteintech Group Inc); F4/80 (27044‐1‐AP; Proteintech Group Inc); CD3 (17617‐1‐AP; Proteintech Group Inc); fibronectin (ab2413; Abcam); Collagen I (#84336; Cell Signaling); Collagen IV (ab6586; Abcam); TGF‐β (#3711; Cell Signaling); PAI‐1 (ab125687; Abcam); α‐SMA (#19245; Cell Signaling); MCP‐1(#2029; Cell Signaling); TNF‐α (ab6671; Abcam); IL‐6 (ab208113; Abcam); ICAM‐1 (ab171123; Abcam); vascular endothelial growth factor (VEGF; 19003‐1‐AP; Proteintech Group Inc); CD31 (#77699; Cell Signaling) and β‐actin (ab8226; Abcam). ELISA kits for MCP‐1 were from ab208979, Abcam.

### 2.2 In vitro studies

Both mouse murine mesangial cells (MC) and tubuloepithelial cells (MCT) were purchased from were ATCC, and authenticated by STR profiling and tested for mycoplasma contamination by GENEWIZ. MC and MCT were cultured in low glucose (5.5 mmol/L D‐glucose) DMEM media supplemented with 100 U/mL penicillin, 100 µg/mL streptomycin and 10% FBS. The transient expression of PTPN2 proteins in cells was performed by adenovirus infection (Ad‐PTPN2 and Ad‐Vehicle; multiplicity of infection 40) for 24 hours before stimulation with HG (30 mmol/L D‐glucose). Protein expressions were analysed by Western blot analyses and ELISA. Cell proliferation was assessed by MTT Cell Proliferation and Cytotoxicity Assay Kit (Beyotime, Shanghai, China) in vitro.

### 2.3 Diabetic model and in vivo experiments

Four‐week‐old male ApoE^‐/‐^ mice were randomly assigned to a control group and diabetic group. The control group was fed a normal diet; the diabetic groups received a high‐fat (HF) diet (34.5% fat, 17.5% protein and 48% carbohydrate; Beijing HFK Bio‐Technology, China). At the age of 10 weeks, intraperitoneal glucose tolerance test (IPGTT) was performed to confirm the appearance of insulin resistance (IR). The control group was injected with citrate buffer intraperitoneally. Those mice showing IR in the diabetic group received a single dose of STZ (Sigma, St. Louis, MO; 75 mg/kg ip in 0.1 mol/L citrate buffer, pH 4.5) intraperitoneally. At the age of 12 weeks, most HF/STZ‐treated mice showed glucose tolerance, IR and hyperglycaemia, as previously reported.^25^ At the age of 12 weeks, the mice in diabetic group with similar degrees of body weight and hyperglycaemia were randomly redivided into vehicle (DM + Vehicle, n = 15) and PTPN2 overexpression (DM + PTPN2, n = 15) groups. The mice received a normal chow were used as non‐diabetic controls, divided into vehicle (N + Vehicle, n = 15) and PTPN2 overexpression (N + PTPN2, n = 15) groups. At the age of 20 weeks, the PTPN2 overexpression group (the DM + PTPN2 group and the N + PTPN2 group) was administered 5 × 10^9^ plaque‐forming units (PFU) of the recombinant pAdxsi adenovirus constitutively expressing PTPN2 by the jugular vein. The vehicle group (the DM + Vehicle group and the N + Vehicle group) received a control empty virus by the jugular vein injection. At the age of 22 weeks, adenovirus transfer was repeated. At the age of 24 weeks, all mice were killed for further biochemical and histological studies. All animal procedures were performed in accordance with animal protocols approved by Shandong University Institutional Animal Care and Use Committee.

### 2.4 Intraperitoneal glucose tolerance test

Intraperitoneal glucose tolerance test (IPGTT) was performed to access glucose tolerance after mice fasted for 12 hours. A bolus of glucose (2 g/kg) was injected intraperitoneally. Blood glucose levels were obtained from the tail vein and measured with the OneTouch Glucometer (LifeScan, Milpitas, CA) at 0, 15, 30, 60 and 120 minutes after injection.

### 2.5 Production of adenoviral vector

The recombinant pAdxsi adenovirus constitutively expressing PTPN2 was constructed using the pAdxsi Adenoviral System (Hanbio Biotechnology Co., Ltd., Shanghai, China). The PTPN2 cDNAs from mouse were inserted into pShuttle‐CMV‐EGFP vector. The pAdxsi vector adenovirus was used as the control vehicle virus. After amplification, viruses were purified, titered and stored at −80°C until used.

### 2.6 Blood and urine examination

At the end of the experiment, the mice were fasted overnight and killed by an overdose of pentobarbital. Fasting blood glucose, total cholesterol (Chol), LDL‐cholesterol (LDL‐chol), HDL‐cholesterol (HDL‐chol), triglyceride (TG) and non‐estesterified fatty acid (NEFA) were measured. Different serum enzymes were estimated following standard methods namely aspartate transaminase (AST) and alanine transaminase (ALT).^26^ Blood urea nitrogen (BUN) and creatinine in the plasma and urinary albumin were estimated using standard kits (Nanjing Jiancheng, Nanjing, China).

### 2.7 Histology and immunohistochemistry

Kidney samples were fixed with 4% paraformaldehyde overnight and embedded in paraffin. Paraffin‐embedded kidney sections (5‐μm thick) were used for histological analysis. Histological scoring (0‐3 scale), glomerular size and mesangial area were quantified in PAS‐stained paraffin sections. Renal fibrosis was examined by polarized light microscopy after Masson staining and Sirius Red staining. Immuno‐detection of proteins (PTPN2, P‐STAT1, P‐STAT3, Arg I, Arg II, CD11c, CD206, Col I, Col IV, Fn, TGF‐β, PAI‐1, α‐SMA, MCP‐1, TNF‐α, IL‐6 and ICAM‐1), T cells (CD3) and macrophages (F4/80) in renal samples was performed by immunohistochemical staining. Positive staining was analysed with Image Pro‐Plus analysis software, and positive area was expressed as a percentage of the total area. Fifteen fields per kidney (×200) were chosen randomly for analysis. Liver tissues were fixed with 4% paraformaldehyde overnight and embedded in paraffin. The slides (5‐μm thick) were stained with HE for histopathological analysis and Sirius Red staining for fibrosis analysis. The vacuolized hepatic cells were quantified in at least 10 sections per mice by the Image J plugins cell counter and expressed as percentage of total cells to analyse liver pathological alteration. Image acquisition was performed on a confocal FV 1000 SPD laser scanning microscope (Olympus, Japan).

### 2.8 Immunofluorescence staining and microscopy

The cryosections were blocked with 5% BSA blocking buffer and stained with antibodies against PTPN2, VEGF and CD31 at 4°C overnight. The stained sections were washed with PBS and further incubated with Alexa Fluor 488 secondary antibodies (Proteintech Group Inc, Chicago, IL) followed by nuclear counterstain (4′,6‐diamidino‐2‐phenylindole). The samples were washed three times in PBS, and examined under a laser scanning confocal microscope (LSM710; Carl Zeiss, Germany), and data were analysed using Image‐Pro Plus 6.0 software (Media Cybernetics).

### 2.9 Western blot analyses

Total proteins from cells and tissues were separated in a 10% SDS‐PAGE and transferred onto PVDF membranes. After blocking in 5% skim milk for 60 minutes, the membranes were incubated with primary antibodies for PTPN2, P‐STAT1, STAT1, P‐STAT3, STAT3, Arg I, Arg II, Col I, Col IV, Fn, TGF‐β, PAI‐1, MCP‐1, TNF‐α, IL‐6, ICAM‐1 and VEGF, followed by anti‐IgG horseradish peroxidase‐conjugated secondary antibody. The membrane bands were visualized by use of chemiluminescence (Millipore) and quantified by densitometry.

### 2.10 Statistical analyses

Values are presented as mean ± SEM. Results were compared by one‐way ANOVA. *P* < 0.05 was considered statistically significant. SPSS 17.0 (SPSS, Chicago, IL) was used for statistical analysis.

## 3 RESULTS

### 3.1 Generation of mouse DN model

**Figure 1:**
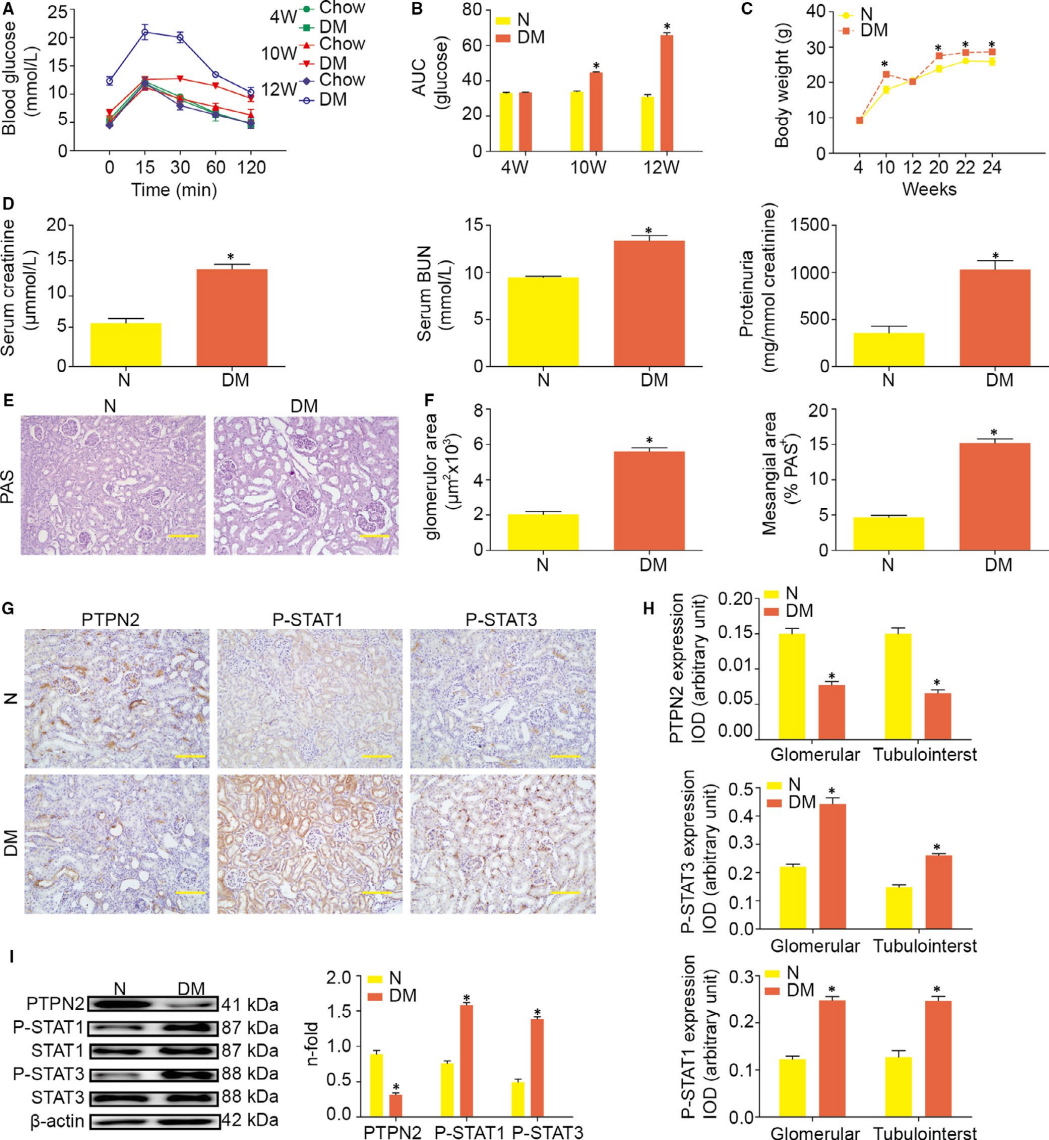
Renal PTPN2 expression decreased after diabetes induction in mice. A, ApoE^‐/‐^ mice were fed on chow diet (Chow) or high‐fat diet (DM) and analysed by intraperitoneal glucose tolerance test (IPGTT) at 4, 10 and 12 weeks of feeding. B, AUC (area under curve) in 4, 10 and 12‐week‐old ApoE^‐/‐^ mice. C, Body weight at the ages of 4, 10, 12, 20, 22 and 24 weeks. D, Serum creatinine, serum BUN (blood urea nitrogen) and proteinuria levels (urine albumin‐to‐creatinine ratio) in ApoE^‐/‐^ mice. E, Representative images of periodic acid‐Schiff (PAS)‐stained kidney sections. F, Glomerular area quantification and PAS + mesangial area analysis in ApoE^‐/‐^ mice. G, Representative micrographs showing positive PTPN2, P‐STAT1, and PSTAT3 immunostaining in glomerular and tubulointerstitium of diabetic mice. H, Quantification of PTPN2, PSTAT1, and P‐STAT3 immunostaining in glomerular and tubulointerstitial compartments. I, Western blot analyses of PTPN2, P‐STAT1 and P‐STAT3 expression in renal cortical lysates from diabetic mice. N: normal; DM: diabetes mellitus; IOD: integrated optical density. Data are mean ± SEM of seven to eight animals per group. *P < 0.05 vs. N. Original magnification, ×200 in E and G

At the age of 4 weeks, the levels of blood glucose between the control and diabetic group were similar (Figure 1A,B). At the age of 10 weeks, IR was induced after a 6‐week HF diet, confirmed by IPGTT (Figure 1A,B). At the age of 12 weeks, the HF/STZ‐treatment resulted in frank hyperglycaemia, glucose tolerance and IR (Figure 1A,B). The mean bodyweight was markedly higher for the diabetic mice than normal diet mice at the age of 4, 10, 20, 22 and 24 weeks (Figure 1C).

Diabetes was associated with renal decline, as demonstrated by an increased serum creatinine, serum BUN and urine albumin‐to‐creatinine ratio (Figure 1D). Histologic assessment of periodic acid‐Schiff (PAS)‐stained kidney samples revealed diabetes increased glomerular size and PAS^+^‐mesangial area (Figure 1E,F).

As shown in Figure 1G, PTPN2 was distributed broadly in glomerular mesangial cells and proximal tubular cells of ApoE^‐/‐^ mice. Moreover, PTPN2 was decreased in the renal cortex of HF/STZ‐induced diabetic mice at the protein level (Figure 1G,I). Consistent with previous study, diabetes led to the activation of STAT signalling pathway, as assessed by STAT1 and STAT3 tyrosine phosphorylation (Figure 1G,I).

Thus, the HF/STZ‐induced diabetic mouse model displayed typical features of hyperglycaemia, glucose tolerance, IR, obesity and renal injury, resembling the state of human DN.

### 3.2 PTPN2 gene therapy protected from diabetes‐associated renal injury in ApoE^‐/‐^ mice

**Figure 2:**
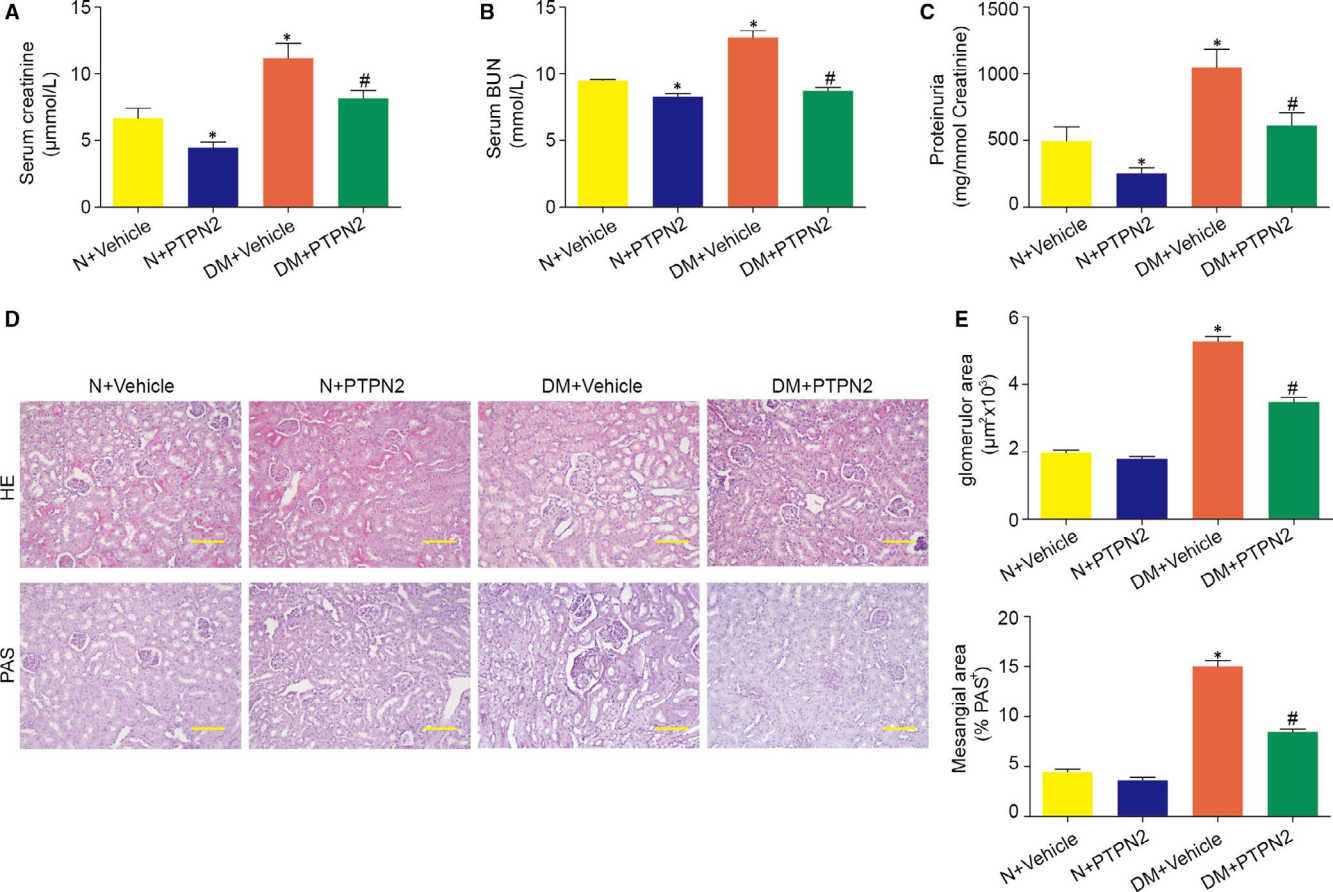
PTPN2 gene therapy protected from diabetes‐associated renal injury in ApoE^‐/‐^ mice. A, Serum creatinine levels in ApoE^‐/‐^ mice with established nephropathy after PTPN2 gene therapy. B, Serum BUN levels in ApoE^‐/‐^ mice with established nephropathy after PTPN2 gene therapy. C, Proteinuria levels in ApoE^‐/‐^ mice with established nephropathy after PTPN2 gene therapy. D, Representative images of HE and PAS staining in renal sections. E, Glomerular area quantification and PAS + mesangial area analysis in the experimental groups. N: normal; DM: diabetes mellitus. Data are mean ± SEM of seven to eight animals per group. *P < 0.05 vs. N + Vehicle; ^#^ *P* < 0.05 vs. DM + Vehicle. Original magnification, ×200 in D

We then investigated the effects of PTPN2 gene therapy on renal dysfunctions in mice with established experimental DN. As shown in Figure 2A,B, PTPN2 gene therapy markedly reduced serum levels of creatinine and BUN in diabetic mice, indicating largely improved renal lesions after PTPN2 gene transfer. Moreover, the proteinuria level, a clinical predictor of renal injury in DN, was significantly ameliorated in diabetic mice after PTPN2 overexpression (Figure 2C). In addition, PTPN2 gene therapy resulted in significant decrease of kidney/body weight ratio (Table 1).

**Table 1:** Metabolic parameters of ApoE^‐/‐^ mice

Variables	N + Vehicle (n = 8)	N + PTPN2 (n = 8)	DM + Vehicle (n = 8)	DM + PTPN2 (n = 8)
Body weight (g)	25.38 ± 0.50	26.44 ± 0.24	29.68 ± 0.55^a^	29.63 ± 0.47
KBWR, g/kg	6.48 ± 0.28	6.51 ± 0.27^a^	14.25 ± 0.57^a^	8.01 ± 0.52^b^
Chol, mmol/L	12.29 ± 1.18	8.32 ± 1.20^a^	23.17 ± 3.33^a^	12.78 ± 1.34^b^
LGL‐chol, mmol/L	1.32 ± 0.17	0.59 ± 0.09^a^	3.35 ± 0.58^a^	1.32 ± 0.23^b^
HDL‐chol, mmol/L	4.66 ± 0.28	3.92 ± 0.48	6.19 ± 0.46^a^	4.78 ± 0.37^b^
TG, mmol/L	0.88 ± 0.10	0.94 ± 0.11	2.08 ± 0.36^a^	0.77 ± 0.09^b^
NEFA, mmol/L	95.00 ± 15.56	108.00 ± 12.71	150.20 ± 14.86^a^	137.50 ± 9.06

Results from the different groups of non‐diabetic and diabetic are reported as mean ± SEM and analysed by two‐way ANOVA followed by Bonferroni post hoc test. Serum sampling was taken under fasting condition and measured.

KBWR, kidney/body weight ratio; Chol, cholesterol; TG, triglyceride; NEFA, non‐estesterified fatty acid.

a P<0.05 vs. N.

b P<0.05 vs. DM.

Histological examination of HE and PAS‐stained kidney samples showed that PTPN2 improved several morphologic changes within the glomerulus (hypercellularity, mesangial matrix expansion and capillary dilation), tubules (atrophy and degeneration) and interstitium (fibrosis and inflammatory infiltrate) of diabetic mice (Figure 2D, Table 2). Digital quantification further indicated that PTPN2 gene therapy reduced glomerular size and PAS^+^‐mesangial area (Figure 2E). Collectively, these data suggested that PTPN2 overexpression effectively attenuated renal injury in experimental DN.

**Table 2:** Renal scores of non‐diabetic and diabetic mice

Histological lesions	N + Vehicle (n = 8)	N + PTPN2 (n = 8)	DM + Vehicle (n = 8)	DM + PTPN2 (n = 8)
Glomerular lesions				
Hypercellularity	0.18 ± 0.12	0.09 ± 0.09	2.09 ± 0.09^a^	0.27 ± 0.14^b^
Mesangial matrix expansion	0.27 ± 0.14	0.18 ± 0.12	2.27 ± 0.14^a^	0.36 ± 0.15^b^
Capillary dilation	0.00 ± 0.00	0.00 ± 0.00	1.72 ± 0.14^a^	0.63 ± 0.15^b^
Tubular lesions				
Degeneration	0.45 ± 0.20	0.36 ± 0.20	2.18 ± 0.12^a^	0.54 ± 0.20^b^
Atrophy	0.36 ± 0.20	0.27 ± 0.14	1.90 ± 0.09^a^	0.63 ± 0.24^b^
Interstitial lesions				
Fibrosis	0.00 ± 0.00	0.00 ± 0.00	1.27 ± 0.14^a^	0.20 ± 0.13^b^
Inflammation	0.18 ± 0.12	0.09 ± 0.09	1.81 ± 0.22^a^	0.45 ± 0.20^b^

PAS‐stained renal samples were semiquantitatively graded (0‐3 scale) in a blinded manner according to the extent of glomerular, tubular, and interstitial damage. Results from the different groups of mice are reported as means ± SEM and analysed by two‐way ANOVA followed by Bonferroni post hoc text.

a P<0.05 vs. N + Vehicle.

b P<0.05 vs. DM + Vehicle.

### 3.3 PTPN2 gene therapy improved insulin resistance and metabolic disorders in diabetic mice

**Figure 3:**
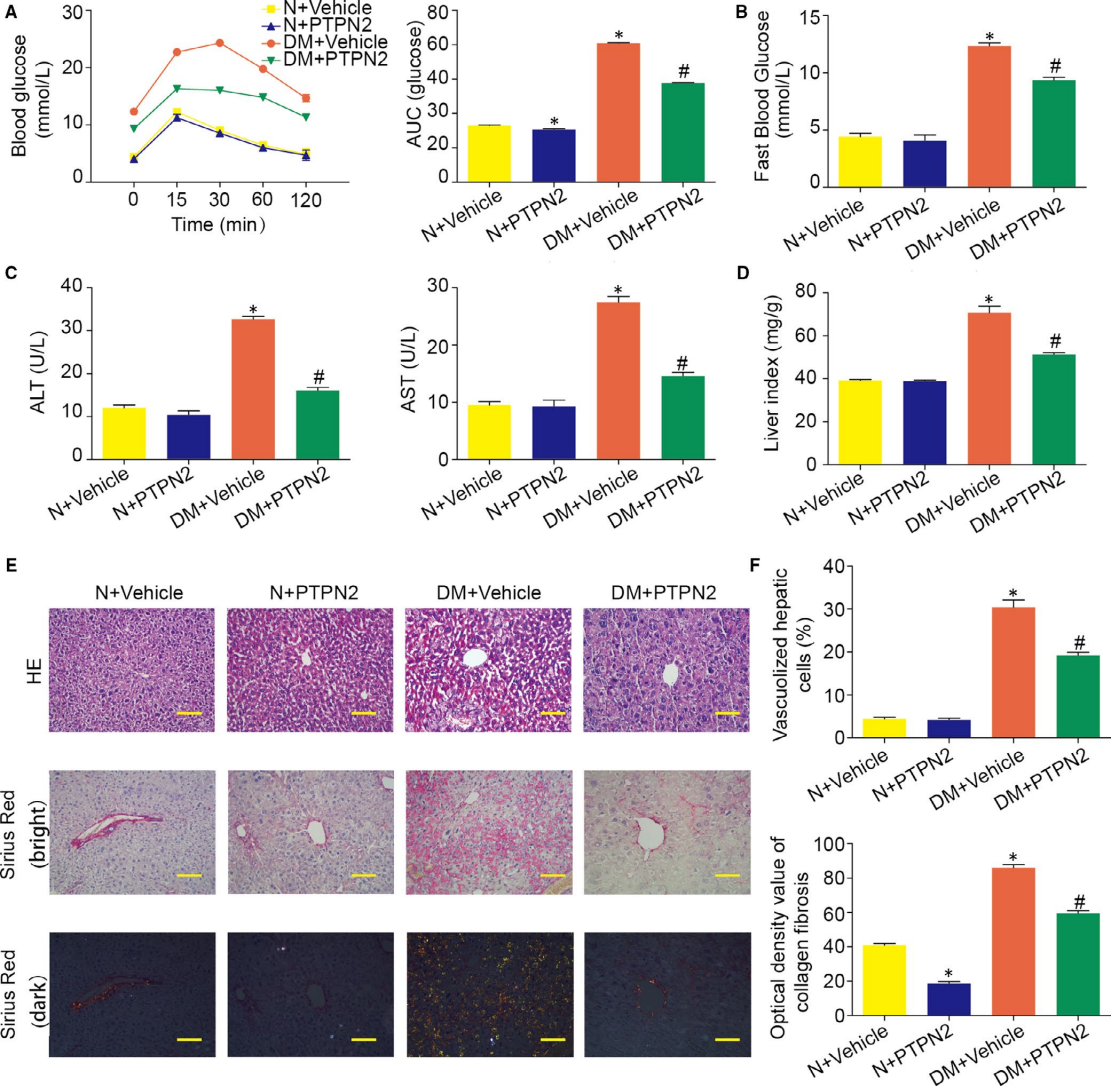
PTPN2 gene therapy improved insulin resistance and metabolic disorder in diabetic mice. A, Glucose tolerance tests and the area under blood glucose concentration curve (AUC) at the age of 24 weeks. B, Fast blood glucose levels at the age of 24 weeks. C, Serum levels of ALT (alanine transaminase) and AST (aspartate transaminase) after PTPN2 gene therapy. D, Liver index of mice with established nephropathy after PTPN2 gene therapy. Liver index = Liver weight (mg)/Body weight (g). E, Representative micrographs of HE and Sirius Red‐sensitive collagen staining to detect liver histopathological alterations. F, Quantification of liver pathological alteration and fibrosis in diabetic mice after PTPN2 gene therapy. N: normal; DM: diabetes mellitus. Data are mean ± SEM of seven to eight animals per group. *P < 0.05 vs. N + Vehicle; ^#^ *P* < 0.05 vs. DM + Vehicle. Original magnification, ×200 in E

It has been well acknowledged that DN is an inflammatory disease triggered by disordered metabolism. Thus, we investigated whether PTPN2 could regulate metabolism in mice with established experimental DN. As shown in Figure 3A,B, PTPN2 gene therapy markedly improved frank hyperglycaemia, glucose tolerance and IR, indicating that better blood glucose control in diabetic mice by PTPN2 overexpression (Figure 3A,B). It has been universally recognized that liver plays a central role in metabolic balance with numerous functions. Thus, we then explored whether liver lesions induced by diabetes were improved after PTPN2 overexpression. As shown in Figure 3C, the increased serum levels of ALT and AST were markedly reduced in diabetic mice after PTPN2 overexpression. In addition, PTPN2 overexpression significantly improved hepatomegaly in diabetic mice, as assessed by the liver index (Figure 3D). Meanwhile, liver lesions induced by diabetes such as hepatic vacuolization, necrosis and fibrosis were significantly improved after PTPN2 gene transfer, as indicated by pathological examination (Figure 3E,F). Furthermore, PTPN2 gene therapy markedly ameliorated disordered metabolism in diabetic mice as demonstrated by serum levels of triglyceride, total cholesterol and LDL‐cholesterol that were decreased to those of control mice (Table 1). These results indicated that PTPN2 gene transfer ameliorated hyperglycaemia and disordered metabolism, which exert a protective effect on DN.

### 3.4 PTPN2 gene therapy reduced diabetes‐induced renal inflammation

**Figure 4:**
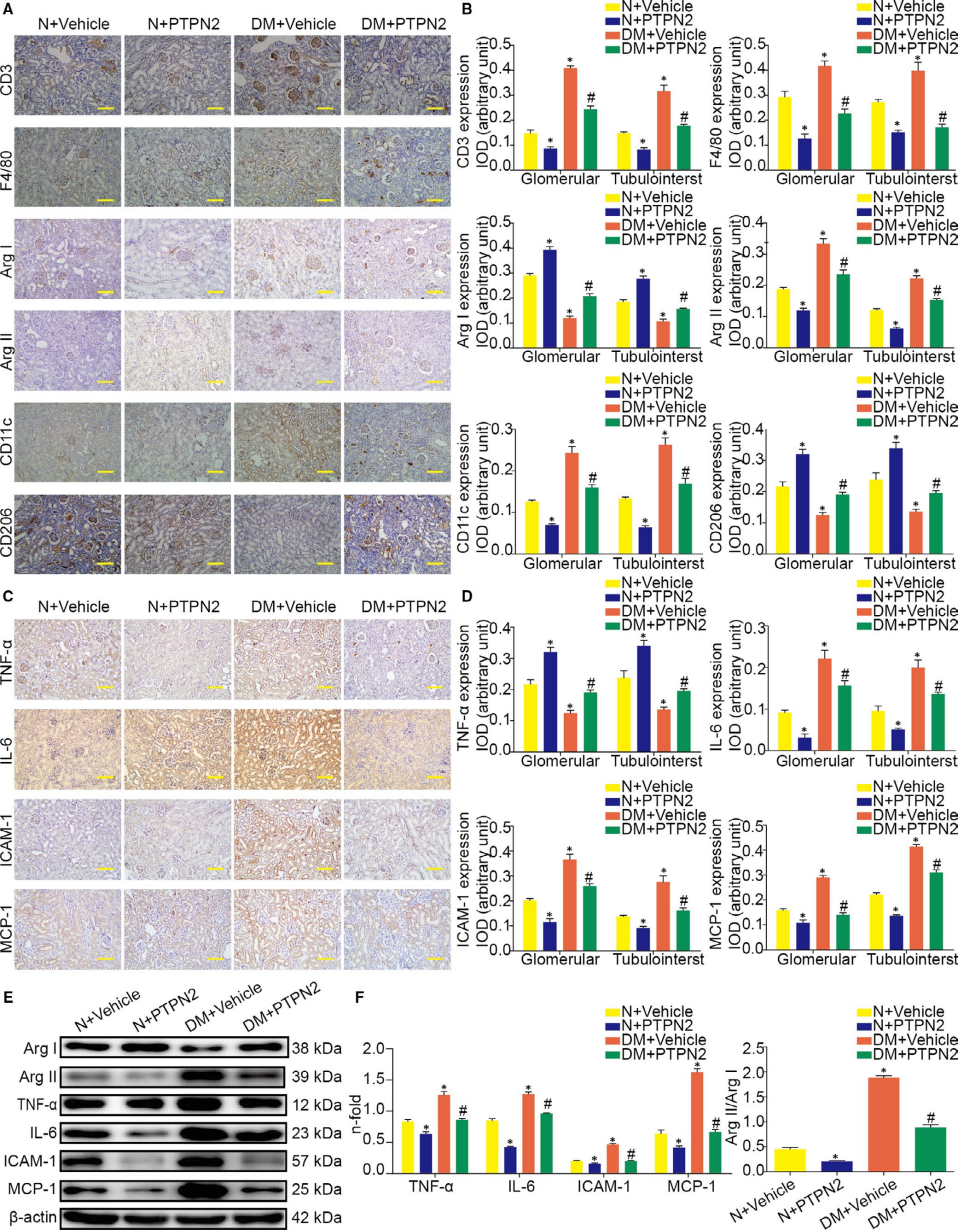
PTPN2 gene therapy reduced diabetes‐induced renal inflammation. A, Representative micrographs showing positive CD3, F4/80, Arg I (Arginase I), Arg II (Arginase II), CD11c and CD206 immunostaining in glomerular and tubulointerstitium. B, Quantification of CD3, F4/80, Arg I, Arg II, CD11c and CD206 immunostaining in glomerular and tubulointerstitial compartments. C, Representative micrographs showing positive TNF‐α (tumor necrosis factor‐α), IL‐6 (interleukin‐6), ICAM‐1 (intercellular cell adhesion molecule‐1) and MCP‐1 (monocyte chemotactic protein 1) immunostaining in glomerular and tubulointerstitium. D, Quantification of TNF‐α, IL‐6, ICAM‐1, and MCP‐1 immunostaining in glomerular and tubulointerstitial compartments. E, Representative Western blot analyses of Arg I, Arg II, TNF‐α, IL‐6, ICAM‐1, and MCP‐1 expression in renal cortical lysates. F, Quantitative analysis of the results in E. IOD: integrated optical density; N: normal; DM: diabetes mellitus; IOD: integrated optical density. Data are mean ± SEM of seven to eight animals per group. *P < 0.05 vs. N + Vehicle; ^#^ *P* < 0.05 vs. DM + Vehicle. Original magnification, ×200 in A and C

The induction of diabetes was associated with the recruitment, retention and activation of leucocytes in the mouse kidney, as indicated by increased expression of leucocyte markers and pro‐inflammatory genes (Figure 4A,C). PTPN2‐treated mice showed a significant decrease in the number of infiltrating CD3^+^ T lymphocytes and F4/80^+^ macrophages (Figure 4A,B). Moreover, PTPN2 gene therapy inhibited the STAT‐dependent genes expression levels of adhesion molecules (ICAM‐1), cytokine (TNF‐α, IL‐6) and monocyte and T‐cell chemokines (MCP‐1) in diabetic kidneys (Figure 4C,E).

To further evaluate whether PTPN2 regulates the functional stage of kidney macrophages, expression levels of CD11c, CD206 and arginase isoforms (Arg II and Arg I) were analysed to distinguish between pro‐inflammatory M1 and anti‐inflammatory M2 phenotypes respectively. Both macrophage phenotypes are present in diabetic kidneys, M1 macrophages (CD11c and Arg II) being the most abundantly expressed in the vehicle group and M2 macrophages (CD206 and Arg I) the predominant macrophage marker in the PTPN2 overexpression group (Figure 4A,E). In summary, these results indicated that PTPN2 gene therapy effectively prevented diabetes‐induced renal inflammation in DN.

### 3.5 PTPN2 gene therapy decreased fibrosis in diabetic mice

**Figure 5:**
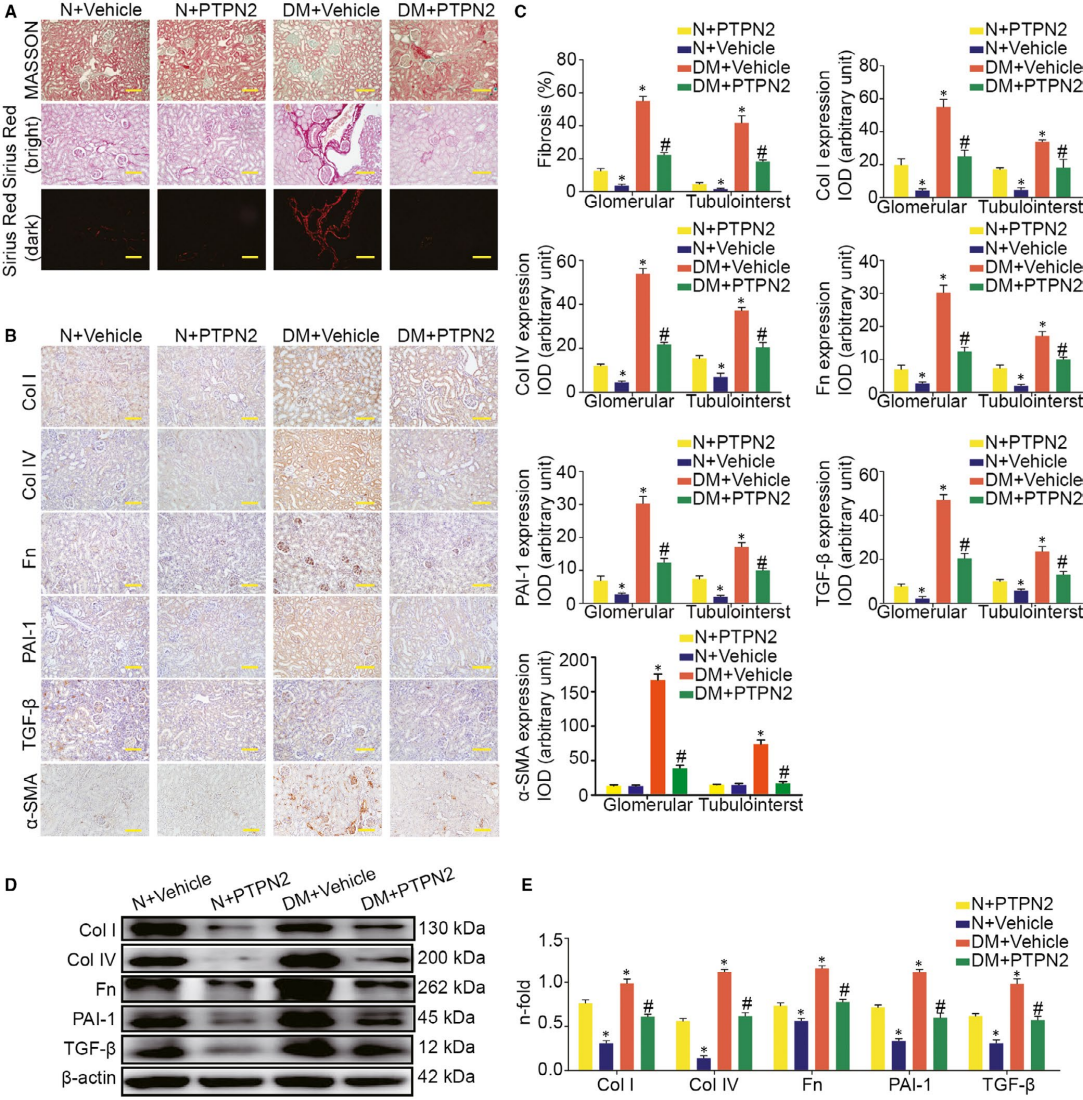
PTPN2 gene therapy decreased diabetes‐induced renal fibrosis. A, Representative images of MASSON, and Sirius Red‐sensitive collagen staining in renal sections. B, Representative micrographs showing positive Col I (collagen I), Col IV (Collagen IV), Fn (fibronectin), PAI‐1 (plasminogen activator inhibitor‐1), and TGF‐β (transforming growth factor‐β) and α‐SMA (α‐smooth muscle actin) immunostaining in glomerular and tubulointerstitium. C, Quantification of fibrosis (% Sirius Red area), Col I, Col IV, Fn, PAI‐1, TGF‐β and α‐SMA immunostaining in glomerular and tubulointerstitial compartments. D, Representative Western blot analyses of Col I, Col IV, Fn, PAI‐1 and TGF‐β expression in renal cortical lysates. E, Quantitative analysis of the results in D. N: normal; DM: diabetes mellitus; IOD: integrated optical density. Data are mean ± SEM of seven to eight animals per group. *P < 0.05 vs. N + Vehicle; ^#^ *P* < 0.05 vs. DM + Vehicle. Original magnification, ×200 in A and B

Since overproduction of extracellular matrix is a hallmark of DN and results in glomerular sclerosis and interstitial fibrosis, we next explored whether PTPN2 could ameliorate renal lesions by preventing renal fibrosis. Diabetic mice showed progressive renal fibrosis with increased total collagen deposition (Figure 5A,C). PTPN2 gene therapy significantly improved renal pathological alterations and collagen accumulation, as assessed by Sirius Red staining and Masson staining (Figure 5A,C, Table 2). In addition, the overexpression of Col I, Col IV, Fn, PAI‐1, TGF‐β and α‐SMA in renal tissue of diabetic mice was markedly attenuated after PTPN2 gene therapy (Figure 5B,C). Quantitative analysis by Western blotting also showed significant decreased protein expressions of Col I, Col IV, Fn, PAI‐1, TGF‐β and α‐SMA in the kidney from diabetic mice after PTPN2 gene therapy (Figure 5D,E). Thus, these results suggested that renal fibrosis was markedly improved after PTPN2 overexpression in DN.

### 3.6 PTPN2 gene therapy inhibited STAT activation in diabetic kidneys

**Figure 6:**
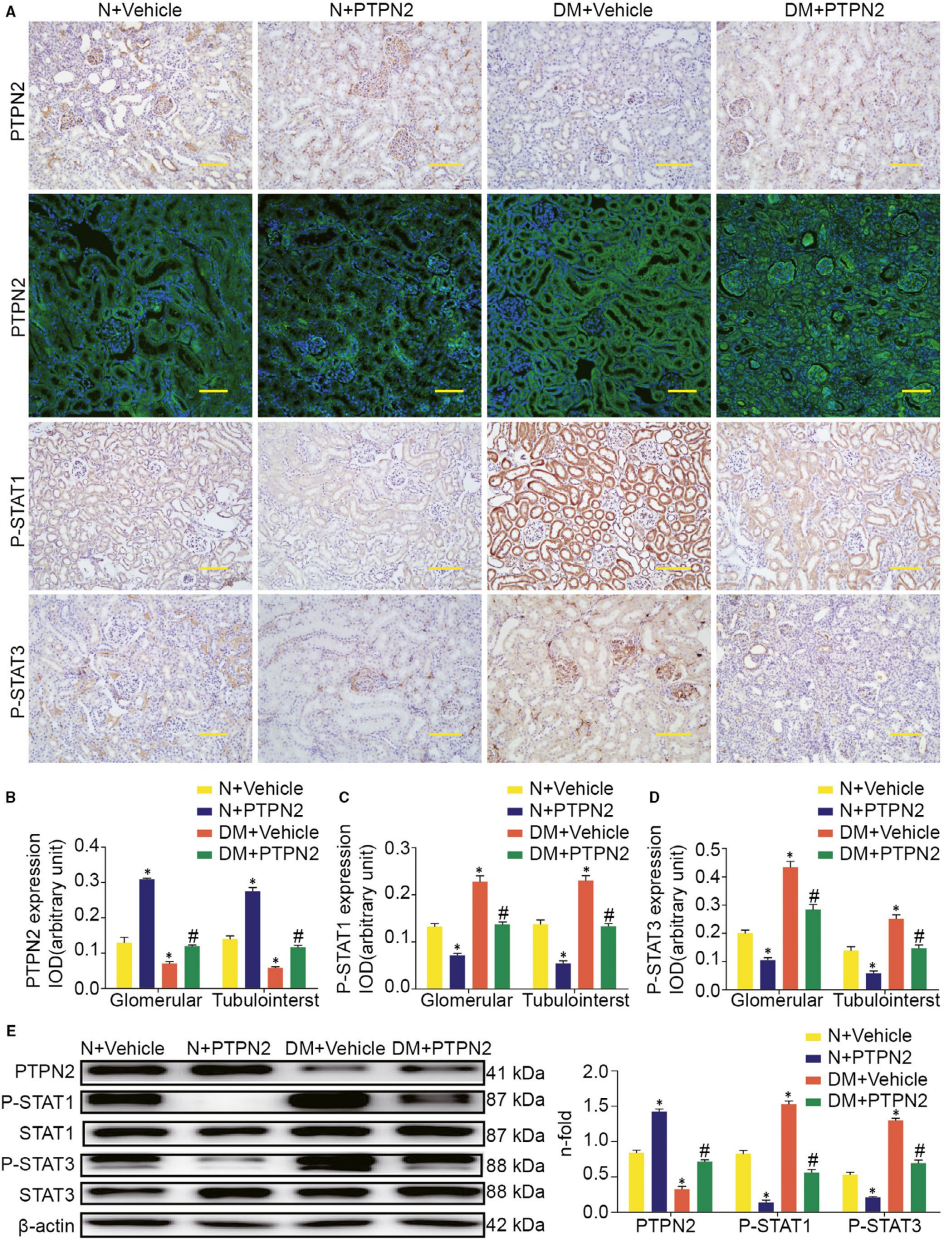
PTPN2 gene therapy inhibited STAT activation in vivo. A, Representative micrographs showing positive PTPN2, P‐STAT1 and P‐STAT3 immunostaining in glomerular and tubulointerstitium. B, Quantification of PTPN2 immunostaining in glomerular and tubulointerstitium. C, Quantification of P‐STAT1 immunostaining in glomerular and tubulointerstitium. D, Quantification of P‐STAT3 immunostaining in glomerular and tubulointerstitium. E, Representative Western blot analyses for PTPN2, P‐STAT1, and P‐STAT3 in renal cortical lysates. N: normal; DM: diabetes mellitus; IOD: integrated optical density. Data are mean ± SEM of seven to eight animals per group. *P < 0.05 vs. N + Vehicle; ^#^ *P* < 0.05 vs. DM + Vehicle. Original magnification, ×200 in A

Mounting evidence has demonstrated that the STAT signalling pathway modulates a broad range of mediators participated in pro‐inflammatory and pro‐fibrotic factors and is an important mechanism through which hyperglycaemia contribute to DN. Thus, we explored whether PTPN2 could modulate STAT activation in DN. As shown in Figure 6A,E, the protein expression of PTPN2 in kidney was markedly increased in the PTPN2 overexpression group. Furthermore, immunohistochemistry to detect the activation status of STAT proteins in the kidney revealed an intense nuclear staining of phosphorylated STAT1 (P‐STAT1) and P‐STAT3 in glomeruli and tubule‐interstitium of diabetic mice receiving vehicle, a significant reduction in PTPN2‐treated mice (Figure 6C,D). Western blot analysis further confirmed diabetic mice treated with Ad‐PTPN2 showed a decrease in the tyrosine phosphorylation of STAT1 and STAT3 (Figure 6E). Our results suggested PTPN2 overexpression inhibited activation of STAT signalling pathway, through which PTPN2 exerted anti‐inflammatory effects in established experimental DN. In summary, these data indicated that PTPN2 gene therapy could exert comprehensive therapeutic effects on DN via improving disordered metabolism and abolishing renal STAT activation.

### 3.7 PTPN2 gene therapy prevented diabetes‐induced renal angiogenesis

**Figure 7:**
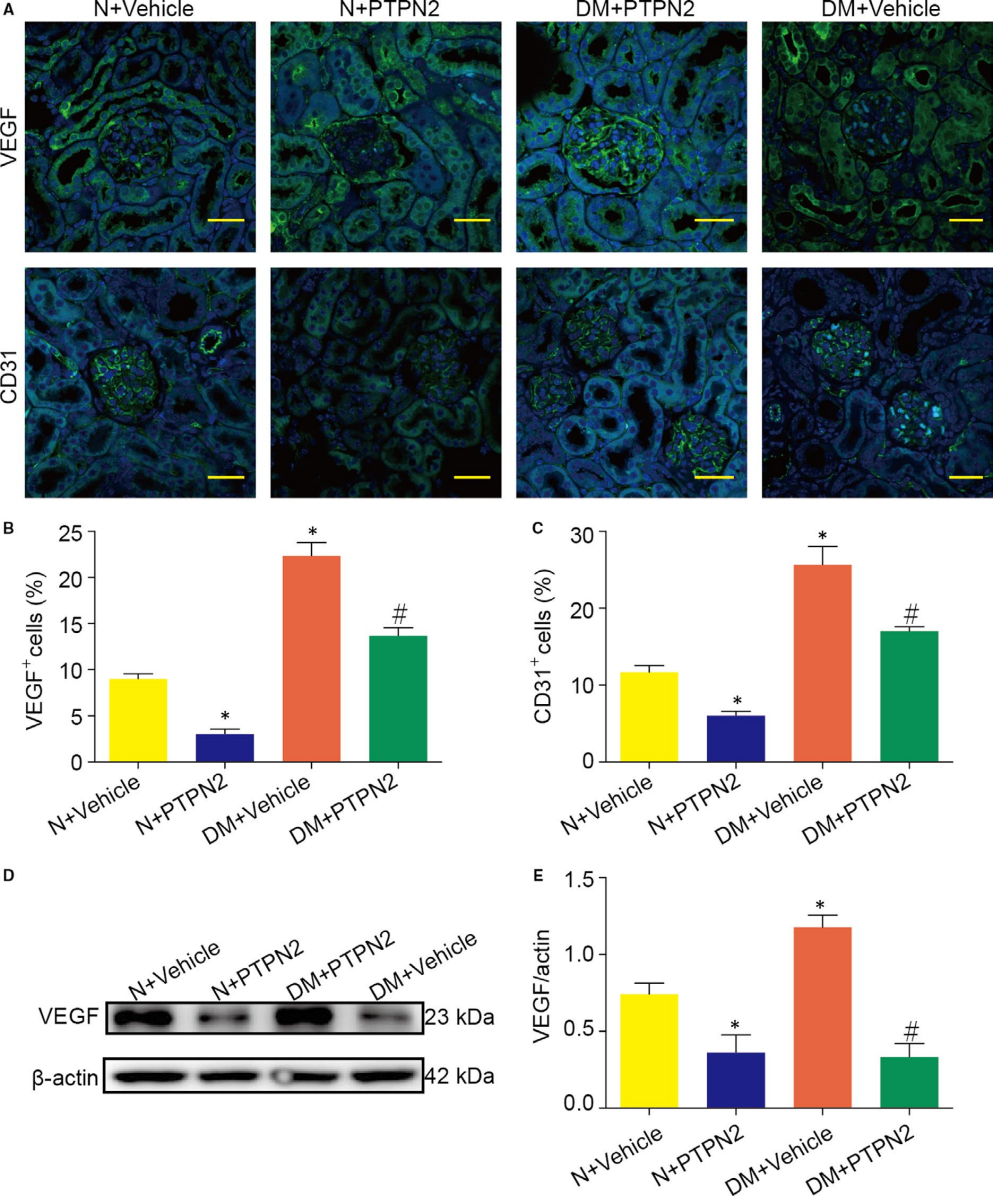
PTPN2 gene therapy prevented diabetes‐induced renal angiogenesis. A, Representative immunofluorescence microscopy of vascular endothelial growth factor (VEGF) and CD31 in peritubular and glomerular regions. B, Quantitative analysis of glomerular VEGF expression. C, Quantitative analysis of glomerular CD31 expression. D, Representative Western blot analyses for VEGF expression in renal cortical lysates. E, Quantitative analysis of the results in D. N: normal; DM: diabetes mellitus. Data are mean ± SEM of seven to eight animals per group. *P < 0.05 vs. N + Vehicle; ^#^ *P* < 0.05 vs. DM + Vehicle. Original magnification, ×400 in A

Abnormal angiogenesis contributes to the formation of new vessels that exert pathological effects on DN. Immunofluorescence staining of CD31 on renal tissue showed increased peritubular capillary formation in diabetic mice (Figure 7A,C). Meanwhile, glomerular capillary formation was also increased in diabetic mice (Figure 7A,C). PTPN2 inhibited the increase of glomerular CD31 expression in diabetic mice (Figure 7A,C). As shown in Figure 7B,D, diabetic mice exhibited increased VEGF expression in renal tissue. PTPN2 overexpression inhibited VEGF expression in established experimental DN (Figure 7B,D). These data indicated that PTPN2 gene therapy could improve DN through abolishing diabetes‐induced renal angiogenesis.

### 3.8 Hyperglycaemia caused PTPN2 down‐regulation and STAT activation in renal cells

**Figure 8:**
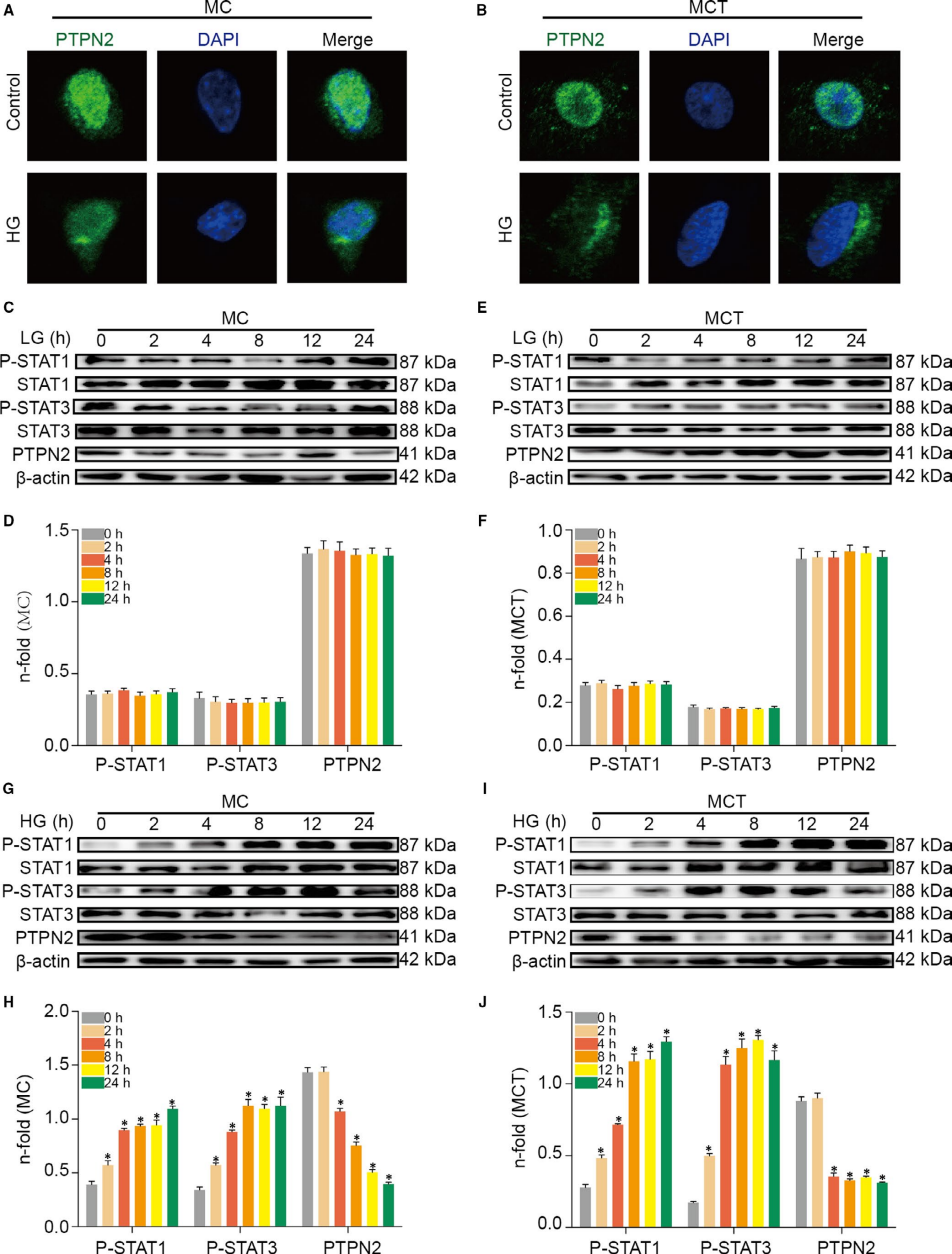
HG caused PTPN2 down‐regulation and STAT activation in cultured renal cells. A, Immunofluorescence microscopy of MC (murine mesangial cells) showed PTPN2 staining in green and nuclear staining in blue. Cells were either left untreated or incubated with high glucose (HG, 30 mmol/L Dglucose) for 24 h. B, Immunofluorescence microscopy of MCT (murine tubuloepithelial cells) showed PTPN2 staining in green and nuclear staining in blue. Cells were either left untreated or incubated with HG for 24 h. C, Representative Western blot analyses showing time course of P‐STAT1, P‐STAT3, and PTPN2 induction by low glucose (LG, 5.5 mmol/L D‐glucose) in MC. D, Quantitative analysis of the results in C. E, Representative Western blot analyses showing time course of P‐STAT1, P‐STAT3, and PTPN2 induction by LG in MCT. F, Quantitative analysis of the results in E. G, Representative Western blot analyses showing time course of P‐STAT1, P‐STAT3 and PTPN2 induction by HG in MC. H, Quantitative analysis of the results in G. I, Representative Western blot analyses showing time course of P‐STAT1, P‐STAT3, and PTPN2 induction by HG in MCT. J, Quantitative analysis of the results in I. LG: low glucose; HG: high glucose. Data are mean ± SEM of three experiments in duplicate. *P < 0.05 vs. Control (0 h)

To corroborate the experimental model we assessed, in vitro, the effect of PTPN2 on murine MC and MCT stimulated with high‐glucose concentrations (HG, medium containing 30 mmol/L D‐glucose) in an attempt to mimic the diabetic milieu. Therefore, we treated the cells with HG for 24 hours and performed immunofluorescence studies. As shown in Figure 8A,B, HG significantly inhibited the expression of PTPN2 in MC and MCT. Incubation of MC and MCT under HG conditions time‐dependently induced the protein expression of PTPN2 compared with low‐glucose conditions (LG, medium containing 5.5 mmol/L D‐glucose) (Figure 8C‐F). Consisting with the immunofluorescence results, immunoblots showed that the protein level of PTPN2 was decreased at 4 hours and then decreased to the lowest level (Figure 8G,I). In addition, the tyrosine phosphorylation of STAT1 and STAT3 was induced by HG, peaking at 24 hours (Figure 8G,I). These findings indicated that hyperglycaemic treatment caused PTPN2 down‐regulation and STAT activation in renal cells.

### 3.9 PTPN2 inhibited HG‐induced STAT activation, STAT‐dependent genes and cell proliferation

**Figure 9:**
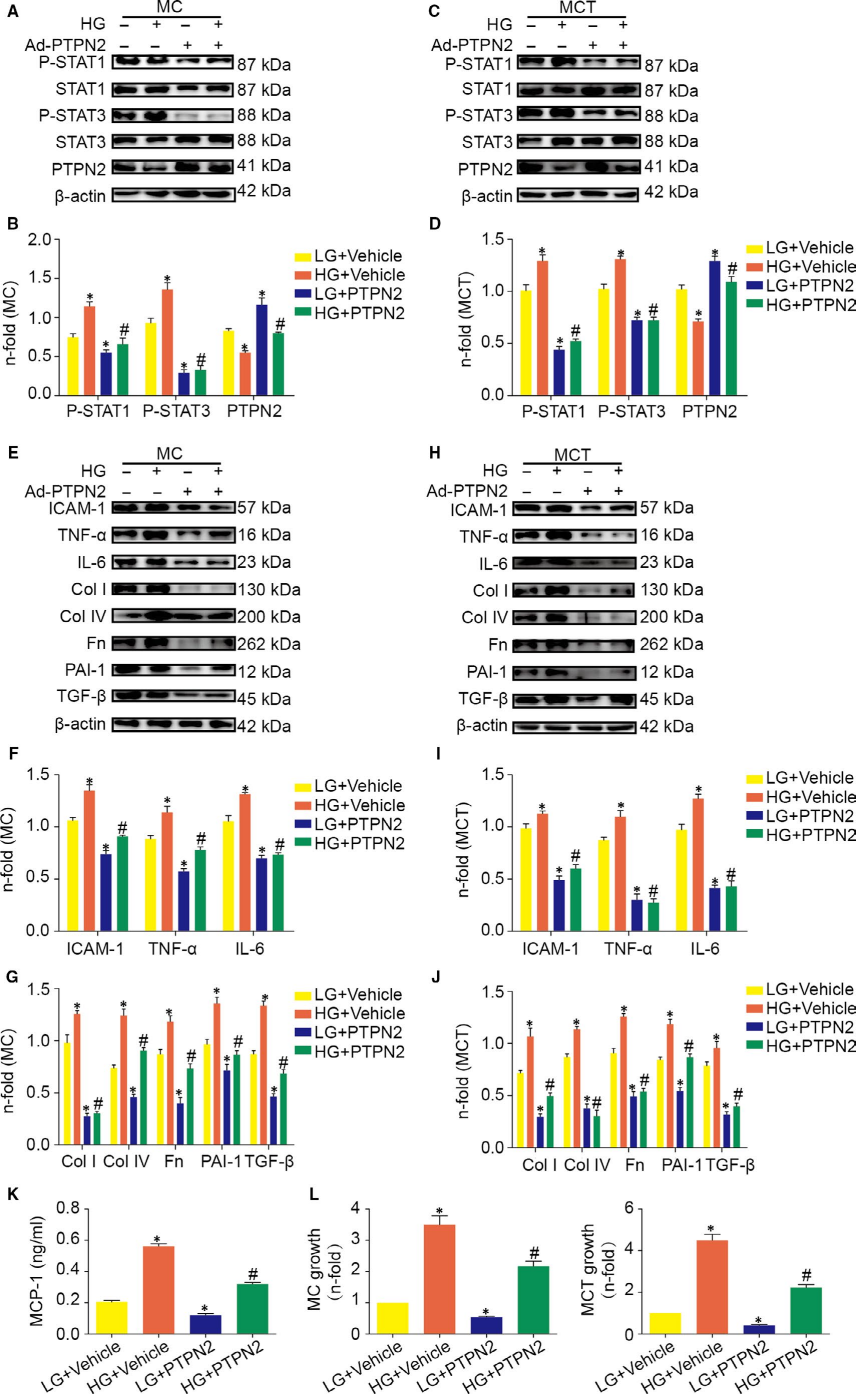
PTPN2 inhibited HG‐induced STAT activation, STAT‐dependent genes, and cell proliferation in vitro. MC and MCT were infected with PTPN2‐expressing adenovirus or control adenovirus. After 24 h, cells were stimulated for an additional 4 h with LG or HG. A, Representative Western blot analyses for P‐STAT1, P‐STAT3 and PTPN2 proteins in total cell extracts from MC. B, Quantitative analysis of the results in A. C, Representative Western blot analyses for P‐STAT1, P‐STAT3 and PTPN2 proteins in total cell extracts from MCT. D, Quantitative analysis of the results in C. E, Representative Western blot analyses for intercellular cell adhesion molecule‐1 (ICAM‐1), tumour necrosis factor‐α (TNF‐α), interleukin‐6 (IL‐6), collagen I (Col I), collagen IV (Col IV), fibronectin (Fn), plasminogen activator inhibitor‐1 (PAI‐1), and transforming growth factor‐β (TGF‐β) proteins in total cell extracts from MC. F, Western blot analyses of ICAM‐1, TNF‐α, and IL‐6 expression in total cell extracts from MC. G, Western blot analyses of Col I, Col IV, Fn, PAI‐1, and TGF‐β expression in total cell extracts from MC. H, Representative Western blot analyses for ICAM‐1, TNF‐α, IL‐6, Col I, Col IV, Fn, PAI‐1 and TGF‐β proteins in total cell extracts from MCT. I, Western blot analyses of ICAM‐1, TNF‐α and IL‐6 expression in total cell extracts from MCT. J, Western blot analyses of Col I, Col IV, Fn, PAI‐1 and TGF‐β expression in total cell extracts from MCT. K, Monocyte chemotactic protein‐1 (MCP‐1) concentration in MC supernatants measured by ELISA. L, Cell proliferation assay in cells transfected with PTPN2‐expressing adenovirus or control adenovirus after 48 h of incubation in LG or HG. LG: low glucose; HG: high glucose. Data are mean ± SEM of three experiments in duplicate. *P < 0.05 vs. LG + Vehicle; ^#^ *P* < 0.05 vs. HG + Vehicle

To investigate the modulation of the STAT pathway by PTPN2 in renal cells, overexpression of PTPN2 protein was induced by adenovirus infection. As shown in Figure 9A‐D, PTPN2 overexpression markedly inhibited STAT1/3 phosphorylation induced by HG in mouse MC and MCT, suggesting that the role of PTPN2 on STAT activation was independent of improved hyperglycaemia.

Among the inflammatory genes induced by HG in renal cells, we investigated the role of PTPN2 on the expression of classical STAT‐dependent genes, including adhesion molecules (ICAM‐1), cytokines (TNF‐α and IL‐6) and chemokines (MCP‐1). As shown in Figure 9E‐H, PTPN2 markedly suppressed the induction of ICAM‐1, TNF‐α and IL‐6 by HG in mouse MC and MCT as compared with control adenovirus (Figure 9F,I). Likewise, PTPN2 overexpression attenuated the secretion of MCP1 induced by HG in MC (Figure 9K). Moreover, treatment with Ad‐PTPN2 prevented the expression of Fn, Col IV, Col I, TGF‐β and PAI‐1, which are pro‐fibrotic genes known to be activated by HG via STAT signalling pathway during DN (Figure 9E‐H).

To evaluate the functional consequences of inflammatory gene reduction, we next examined the effect of PTPN2 on cell proliferation‐important processes involved in renal damage during DN. As shown in Figure 9L, the proliferative effect of HG on MC and MCT was prevented by PTPN2 overexpression.

These data suggested that PTPN2 could inhibit HG‐induced STAT activation, STAT‐dependent genes and cell proliferation in murine mesangial cells and tubuloepithelial cells.

## 4 DISCUSSION

In this study, we found that diabetic mice developed albuminuria, mesangial matrix expansion, tubulointerstitial fibrosis and macrophage influx and showed a decreased renal function; PTPN2 was markedly down‐regulated in diabetic mice, with increased activation of STAT and STAT‐dependent pro‐inflammatory and pro‐fibrogenic cytokines; PTPN2 gene therapy could exert protective effects on DN via ameliorating metabolic disorders and inhibiting renal micro‐inflammation.

Chronic micro‐inflammation is the common pathway for promoting the development and progression of DN, and anti‐inflammatory therapy would exert protective effects on DN.^27^, ^28^ We found that HG could induce the activation of mesangial and tubular cells, which lead to expression of adhesion molecules (ICAM‐1), pro‐inflammatory cytokines (TNF‐α and IL‐6) and chemo‐attractant cytokines (MCP‐1). In turn, these molecules are key mediators of renal lesions by recruiting circulating leucocytes (T lymphocytes and classic M1 pro‐inflammatory macrophages) and facilitating transmigration of these cells into renal tissue. Moreover, these infiltrating leucocytes are also a source of cytokines and other mediators which amplify and perpetuate the inflammatory reaction in kidney, finally contributing to the development and progression of DN. Thus, ameliorating metabolic disorder and decreasing micro‐inflammation level may be crucial for improving renal injury and fibrosis.

PTPN2 stands at the crossroad of multiple signalling mechanisms and has emerged as an interesting therapeutic target with regulation of metabolism and micro‐inflammation.^13^, ^15^, ^18^, ^29^, ^30^ Recent studies have suggested an emerging role of PTPN2 in disordered metabolism. Liver‐specific PTPN2 deficiency led to the development of metabolic syndrome.^13^, ^15^ Consisting with these studies, our results suggested that PTPN2 overexpression ameliorated hyperglycaemia and improved serum levels of triglyceride, total cholesterol and LDL‐cholesterol in HF/STZ‐induced diabetic mice, further uncovering the biological function of PTPN2 in metabolism modulation. It has been universally recognized that simply targeting systemic hyperglycaemia and metabolic disorders is not sufficient to arrest the progression of DN, indicating that there must be other molecular mechanism responsible for therapeutic effects of PTPN2 for DN. In addition to the tight control of hyperglycaemia and metabolic disorders, anti‐inflammation has been considered to be a therapeutic approach to minimize the diabetic complications. We found PTPN2 expression was suppressed in kidney of diabetic mice and correlated with renal injury in diabetes. Evidence is emerging for the involvement of PTPN2 in inflammatory diseases (eg Crohn's disease, T1DM and rheumatoid arthritis).^16^, ^24^, ^31^ Consistent with these results, we demonstrated that PTPN2 suppressed renal injury and fibrosis via down‐regulation of pro‐inflammatory cytokines and subsequent lymphocytes influx. Thus, our study demonstrates that PTPN2 could ameliorate renal injury and fibrosis via regulation of metabolism disorders and micro‐inflammation. However, the downstream mediator of PTPN2 in modulating micro‐inflammation during diabetes remains unknown.

STAT signalling pathway is an important downstream mediator of PTPN2^17^ and exerts many biological functions, such as anti‐inflammatory and anti‐fibrotic actions in acute and chronic inflammatory diseases.^21^, ^22^, ^32^, ^33^ Previous studies have demonstrated that suppression of PTPN2 expression contributing to sustained STAT activation leads to the development and progression of chronic inflammatory diseases.^14^, ^29^ Consistent with previous data, we found that PTPN2 overexpression could inhibit STAT1/3 activation in vitro and in vivo. Therefore, we proposed that PTPN2 gene therapy may reduce the expression of pro‐inflammatory and pro‐fibrotic cytokines via inhibiting STAT1/3 signalling pathway and thus improve renal lesions and fibrosis in DN.

Moreover, our results on the anti‐angiogenic effects of PTPN2 also interpreted the amelioration of renal injury in diabetic mice. VEGF‐induced angiogenesis contributes to the pathogenesis of DN^34^, ^35^ and antibodies directed against VEGF have been shown to prevent proteinuria and glomerular hypertrophy in animal models of diabetes.^36^, ^37^ Previous studies demonstrated that activation of PTPN2 contributes to the suppression of VEGF‐induced endothelial cell proliferation and angiogenic sprouting.^38^ Consistent with these results, we found that angiogenesis using CD31 as marker was significantly increased in kidney in the diabetic group. PTPN2 gene therapy significantly suppressed VEGF expression and angiogenesis in diabetic mice. The immature structure and function of neo‐vascular increase glomerular filtration barrier and elevate permeability, resulting in 24‐hour proteinuria, glomerular sclerosis, interstitial fibrosis by promoting extracellular matrix deposition.^34^, ^39^

In summary, PTPN2, as a critical regulator for metabolic disorder and inflammation, participated in DN. The kidney protective roles with PTPN2 overexpression indicate a potential role for PTPN2 analogue in treating DN.

## CONFLICT OF INTEREST

No potential conflict of interest relevant to this article were reported.
